# Protective role of NRF2 in macrovascular complications of diabetes

**DOI:** 10.1111/jcmm.15583

**Published:** 2020-07-06

**Authors:** Junduo Wu, Xiaodan Sun, Ziping Jiang, Jun Jiang, Linlin Xu, Ao Tian, Xuechun Sun, Huali Meng, Ying Li, Wenlin Huang, Ye Jia, Hao Wu

**Affiliations:** ^1^ Department of Cardiology The Second Hospital of Jilin University Changchun China; ^2^ Intensive Care Unit The Second Hospital Cheeloo College of Medicine Shandong University Jinan China; ^3^ Department of Hand and Foot Surgery The First Hospital of Jilin University Changchun China; ^4^ Department of Neurosurgery The Second Hospital Cheeloo College of Medicine Shandong University Jinan China; ^5^ Department of Neurology The Second Hospital Cheeloo College of Medicine Shandong University Jinan China; ^6^ Department of Nutrition and Food Hygiene School of Public Health Cheeloo College of Medicine Shandong University Jinan China; ^7^ Department of Dermatology Affiliated Hospital of Beihua University Jilin China; ^8^ School of Science and Technology Georgia Gwinnett College Lawrenceville GA USA; ^9^ Beckman Research Institute of City of Hope Duarte CA USA

**Keywords:** complications, diabetes, macrovascular, NRF2, oxidative stress

## Abstract

Macrovascular complications develop in over a half of the diabetic individuals, resulting in high morbidity and mortality. This poses a severe threat to public health and a heavy burden to social economy. It is therefore important to develop effective approaches to prevent or slow down the pathogenesis and progression of macrovascular complications of diabetes (MCD). Oxidative stress is a major contributor to MCD. Nuclear factor (erythroid‐derived 2)‐like 2 (NRF2) governs cellular antioxidant defence system by activating the transcription of various antioxidant genes, combating diabetes‐induced oxidative stress. Accumulating experimental evidence has demonstrated that NRF2 activation protects against MCD. Structural inhibition of Kelch‐like ECH‐associated protein 1 (KEAP1) is a canonical way to activate NRF2. More recently, novel approaches, such as activation of the *Nfe2l2* gene transcription, decreasing KEAP1 protein level by microRNA‐induced degradation of *Keap1* mRNA, prevention of proteasomal degradation of NRF2 protein and modulation of other upstream regulators of NRF2, have emerged in prevention of MCD. This review provides a brief introduction of the pathophysiology of MCD and the role of oxidative stress in the pathogenesis of MCD. By reviewing previous work on the activation of NRF2 in MCD, we summarize strategies to activate NRF2, providing clues for future intervention of MCD. Controversies over NRF2 activation and future perspectives are also provided in this review.

## 1 INTRODUCTION

Diabetes is predicted to be the seventh leading cause of death in the world in 2030.^1^, ^2^ Macrovascular complications of diabetes (MCD)—basically ischaemic heart disease, cerebrovascular disease and peripheral vascular disease—develop in over a half of the diabetic population, resulting in high morbidity and mortality.^3^ This poses a severe threat to the world's public health. Despite the successful control of hyperglycaemia, hypertension and hyperlipidaemia, the diabetic patients are still at risk of developing MCD.^4^ Therefore, it is crucial to identify more viable drug targets and develop more effective approaches, in order to prevent or slow down the pathogenesis and progression of MCD.

Oxidative stress is a key mechanism by which diabetes induces its complications.^5^ Under diabetic condition, excessive reactive oxygen species (ROS) are produced, causing detrimental cellular events, such as formation of advanced glycation end products (AGEs) and overexpression of receptor for AGEs (RAGE), along with activation of polyol pathway, hexosamine pathway and protein kinase C (PKC).^5^, ^6^ These contribute to the pathogenesis and progression of MCD.

The transcription factor nuclear factor (erythroid‐derived 2)‐like 2 (NRF2) plays a critical role in cellular defence against oxidative stress.^7^ NRF2 turns on the transcription of various antioxidant genes, producing cellular antioxidants ^7^ that act as scavengers of free radicals and prevent the oxidative stress‐driven pathogenesis of MCD. NRF2 is negatively regulated by Kelch‐like ECH‐associated protein 1 (KEAP1) in the cytoplasm. KEAP1 restricts NRF2 from nuclear translocation on one hand and facilitates proteasomal degradation of NRF2 on the other.^8^ Therefore, small molecule‐induced structural inhibition of KEAP1 protein—a canonical way to activate NRF2—has become a research hotspot in the past two decades, with the protective outcome verified in animal models of MCD.

In addition to structural inhibition of KEAP1 protein, activation of NRF2 in MCD has been achieved through other approaches. These include activation of *Nfe2l2* gene transcription, decrease in KEAP1 protein level by microRNA‐induced degradation of *Keap1* mRNA, prevention of proteasomal degradation of NRF2 protein and modulation of other upstream regulators of NRF2. Taken together, these investigations have provided alternative strategies to activate NRF2, and shed light upon novel targets upstream of NRF2 for the intervention of MCD as well.

In this review, we summarize and discuss the various strategies arisen to activate NRF2 and their outcome in MCD, with the aim of providing insights into future management of MCD.

## 2 PATHOPHYSIOLOGY OF MCD

**FIGURE 1:**
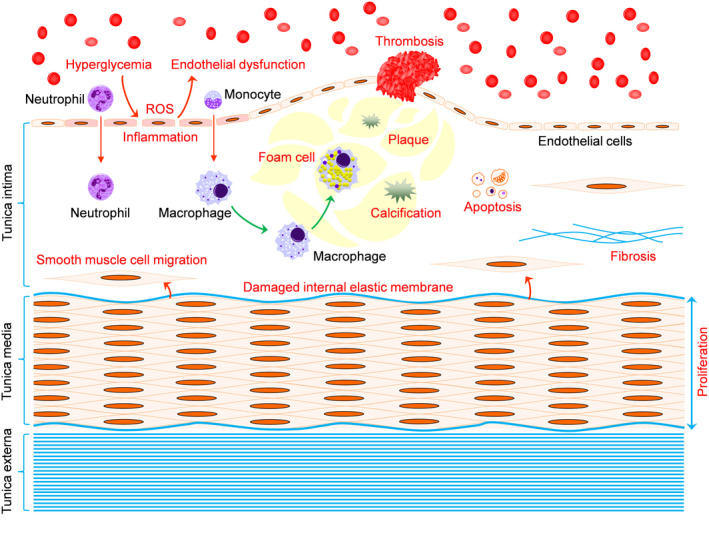
Pathophysiology of macrovascular complications of diabetes (MCD). Hyperglycaemia causes formation of reactive oxygen species (ROS) and inflammation in the endothelium, leading to endothelial dysfunction as a critical first step towards MCD. Under diabetic condition, the permeability of the inflamed endothelium is increased, allowing recruitment of neutrophils and monocytes into the tunica intima, where the macrophages—differentiated from monocytes—engulf lipids and become foam cells that gradually form a plaque. Calcium can be deposited in the plaque. Thrombus forms at the location where the plaque breaks. Apoptosis is induced, and fibrosis is accumulated. Smooth muscle cells proliferate, thereby thickening the tunica media. Smooth muscle cells can migrate into tunica intima through damaged internal elastic membrane, contributing to atherogenesis. Red characters, detrimental processes resulting in atherosclerosis

The diabetes‐driven atherosclerosis is the main cause of MCD. The typical pathological features of atherosclerosis in arteries are characterized by generation of fibrosis, proliferation and structural derangement of smooth muscle cells, thickening of tunica media, accumulation of lipid, formation of plaque, calcification and thrombosis.^9^ Under microscope, adhesion of blood cells to the endothelium and extension of interendothelial connection may be observed, indicating the diabetes‐enhanced adhesion and permeability of the endothelium.^10^ The arterial pathophysiological events induced by diabetes (Figure 1) eventually lead to vascular dysfunction and ischaemic complications.

## 3 ROLE OF OXIDATIVE STRESS IN THE PATHOGENESIS OF MCD

Oxidative stress reflects an imbalance between the status of ROS and the antioxidant ability of a biological system. Diabetes induces generation of ROS through formation of AGEs, overexpression of RAGE and activation of polyol pathway, hexosamine pathway and PKC.^5^ Upon diabetes, the excessive ROS exceed the scavenging capacity of the cellular antioxidant system, resulting in damage to proteins, lipids and DNAs.^11^ Moreover, the diabetes‐induced ROS provokes inflammation, which in turn exacerbates oxidative stress. This vicious circle formed by ROS and inflammation ^12^ contributes to fibrosis and calcification in the plaque,^13^, ^14^ during a later stage of atherosclerosis.

Endothelial dysfunction is a critical pathophysiological event prior to MCD, with oxidative stress and inflammation as major contributors (Figure 1).^15^ Once formed, typical pathological features of atherosclerosis—such as fibrosis, tunica media thickening, plaque and calcification—are impossible to be reversed. It is therefore crucial to improve diabetes‐induced endothelial dysfunction, the effect of which may efficiently prevent or slow down atherogenesis. In this regard, targeting oxidative stress is a viable strategy.

## 4 PROTECTIVE EFFECTS OF NRF2 ON MCD

As supplementation of antioxidants, such as vitamin E, vitamin C, coenzyme Q10, alpha‐lipoic acid, L‐carnitine and ruboxistaurin, has proven non‐beneficial to diabetic complications,^16^ attention has been transferred to activation of the inner cellular antioxidant capacity. NRF2—the governor of the cellular antioxidant defence system—activates the transcription of downstream antioxidant genes by binding antioxidant response element in the promoter regions of the antioxidant genes, such as haem oxygenase 1 (*Hmox1*), NAD(P)H dehydrogenase (quinone 1) (*Nqo1*), glutathione (*Gsh*), superoxide dismutase (*Sod*), gamma‐glutamylcysteine synthetase and catalase (*Cat*).^17^, ^18^, ^19^

NRF2 is negatively regulated by KEAP1.^7^, ^8^, ^20^, ^21^ Oxidative stress disrupts critical cysteine residues in KEAP1, resulting in the release of NRF2 from the KEAP1‐NRF2 complex.^22^ Hence, a short‐term oxidative stress generated upon diabetes activates NRF2 as a compensatory protective mechanism through which the cells protect against hyperglycaemia‐induced injuries.^23^ However, accumulating evidence has demonstrated that cardiovascular NRF2 antioxidant signalling is impaired after a long‐term exposure to hyperglycaemia.^24^, ^25^, ^26^, ^27^, ^28^, ^29^ This decompensatory effect exposes the cells to more severe injuries. Thus, activation of NRF2 is beneficial to the vasculature under both short‐term and long‐term diabetic conditions prior to atherosclerosis, especially the latter.

To date, many studies have reported the protective effects of NRF2 activation on diabetic complications, such as diabetic cardiomyopathy, nephropathy and retinopathy. However, less is known for the impact of NRF2 activation on MCD, as summarized below.

### 4.1 Canonical structural inhibition of KEAP1 in MCD

#### 4.1.1 Sulforaphane (SFN)

SFN derives from broccoli sprouts^30^ and modifies specific cysteine residues in KEAP1 protein, thereby changing its conformation. This enables NRF2 to dissociate from KEAP1, promoting NRF2 nuclear translocation and preventing proteasomal degradation of NRF2.^31^

The beneficial effects of SFN on MCD have been reported.^32^ In a mouse model of type 2 diabetes, SFN activated NRF2 antioxidant signalling in the aorta and attenuated the diabetes‐induced oxidative stress, inflammation, apoptosis, cell proliferation, thickening of the tunica media and accumulation of collagen in the aorta.^32^ In an animal model of non‐obese type 2 diabetes (Goto‐Kakizaki rats), SFN reversed the diabetes‐repressed expression of aortic NRF2, attenuated the production of aortic O_2_ and AGEs and improved nitric oxide (NO)‐dependent and nitric oxide nitric oxide‐independent vasorelaxation.^29^

Moreover, SFN inhibits nuclear factor‐kappa B (NF‐κB)^33^—a key pro‐inflammatory factor in diabetes‐induced vascular inflammation. Crosstalk may exist between NRF2 and NF‐κB. HO‐1, a potent antioxidant downstream of NRF2, has been shown to repress NF‐κB activity by reducing cellular labile iron content.^34^ NF‐κB, in turn, suppresses NRF2‐induced HO1 production.^35^ The predominant activity of NF‐κB under diabetes might explain the impaired NRF2 antioxidant activity in the vasculature under a long‐term diabetic condition. Given that NF‐κB is a transcription factor, it would be interesting to explore the effect of NF‐κB on the expression of *Keap1* in further studies.

The finding that SFN improves insulin resistance in patients with type 2 diabetes^36^ suggests that SFN may prevent MCD at much earlier stages including diabetes and obesity.

#### 4.1.2 Dh404

Dh404 is a derivative of bardoxolone methyl. The latter was tested in clinical trials for treatment of diabetic nephropathy (DN).^37^ Dh404 activates NRF2 via modification of KEAP1,^38^ a mechanism similar to SFN. Tan et al showed that Dh404 lessened diabetes‐induced atherosclerosis with reduction in oxidative stress and inflammatory factors at lower (3 and 10 mg/kg/d) doses in streptozotocin (STZ)‐induced diabetic apolipoprotein E knockout mice.^38^ However, at a higher dose (20 mg/kg/d), Dh404 increased the expression of pro‐inflammatory mediators monocyte chemoattractant protein‐1 and NF‐κB in the kidney. This side effect of the higher dose needs to be further investigated. Because the expression of the NRF2 downstream genes was not further enhanced in the higher‐dose group compared with the lower‐dose group, it is insufficient to conclude that a drastic activation of NRF2 has detrimental effects. Instead, other mechanisms might account for Dh404's toxicity. Further studies are needed to determine the pharmacological activities of dh404.

#### 4.1.3 Dimethyl fumarate (DMF, BG‐12)

DMF is a known NRF2 activator and has been used for clinical treatment of multiple sclerosis.^39^ Ha et al researched the effect of DMF on vascular calcification in vascular smooth muscle cells (VSMCs) and aortas of C57BL/6J mice.^40^ DMF activated NRF2, attenuating calcification in both the VSMCs and aortas. Moreover, this effect of DMF was abolished when NRF2 was knocked down in VSMCs.^40^ This study may indicate a potential protective effect of DMF on MCD. The approval of DMF in clinical use has granted DMF a unique advantage in its potential application in MCD.

#### 4.1.4 Tert‐butyl hydroquinone (tBHQ)

tBHQ activates NRF2 by targeting Cys‐151 within KEAP1 protein.^41^ tBHQ was reported to ameliorate diabetes‐driven atherosclerosis in apolipoprotein E‐deficient mice.^42^ In this study, tBHQ was found to enhance NRF2 activity in macrophages and VSMCs within atherosclerotic lesions, promoting autophagic activity. This led to decrease in size, extension and lipid content of the atheroma plaques, as well as reduction of lesional macrophages, foam cell size and chemokine expression.^42^

### 4.2 Non‐canonical ways to activate NRF2 in MCD

In recent years, strategies towards NRF2 activation in MCD have evolved from structural inhibition of KEAP1 to other regulatory mechanisms, shedding light upon novel targets and approaches for the intervention of MCD.

#### 4.2.1 Targeting *Keap1* mRNA

##### MicroRNA‐200a (miR‐200a)/*Keap1* mRNA

We and others have reported that miR‐200a targets *Keap1* mRNA, leading to degradation of *Keap1* mRNA and activation of NRF2.^24^, ^43^, ^44^ Recently, our group found that the miR‐200a/*Keap1*/NRF2 axis played an essential role in protecting against diabetes‐induced endothelial dysfunction.^45^ In aortic ECs isolated from C57BL/6 wild‐type mice, miR‐200a mimic or inhibitor modulated KEAP1/NRF2 antioxidant signalling and manipulated oxidative stress and inflammation under high glucose (HG) condition. These effects were completely abrogated by knockdown of *Keap1*, indicating that *Keap1* mRNA is a major target of miR‐200a. Moreover, the protective effect of miR‐200a mimic on HG‐induced endothelial oxidative stress and inflammation was completely abolished in aortic ECs isolated from C57BL/6 *Nfe2l2* knockout mice, suggesting that NRF2 is required for miR‐200a's actions. Further, supplementation of miR‐200a inhibited aortic *Keap1* expression, activated NRF2 signalling and attenuated hyperglycaemia‐induced oxidative stress, inflammation and endothelial dysfunction in the wild‐type, but not *Nfe2l2* knockout, mice.^45^ These findings have verified that domestic miR‐200a/KEAP1/NRF2 directly controls endothelial antioxidant activity, providing miR‐200a and *Keap1* mRNA as viable targets for the intervention of MCD.

MiR‐200a/KEAP1/NRF2 not only provides direct protection to the endothelium, but also benefits the endothelium through a remote route by its regulation of fibroblast growth factor 21 (FGF21). FGF21 regulates glucose and lipid metabolism and has potential for the treatment of diabetes.^46^ FGF21 was shown to prevent aortic pathologies in STZ‐induced diabetic mice.^46^ In OVE26 type 1 diabetic mice, *Fgf21* mRNA and protein levels were found to be increased in the liver and plasma, leading to protection against the diabetes‐induced aortic fibrosis and inflammation.^47^ Further investigation showed that inhibition of HDAC3 with RGFP‐966 enhanced hepatic miR‐200a expression, the effect of which decreased *Keap1* mRNA and protein levels, thereby promoting NRF2‐mediated *Fgf21* gene expression. FGF21, produced in the liver, was then released to the plasma and protected the diabetic aorta.^47^

##### MicroRNA‐24 (MiR‐24)/*Keap1* mRNA

Endothelial repair after stent implantation is delayed in diabetic patients compared with that in non‐diabetic patients.^48^ MiR‐24, which targets *Keap1* mRNA,^44^ was found to activate NRF2/HO1 signalling, restore SOD and GSH, and suppress ROS and malondialdehyde production in HG‐stimulated VSMCs.^48^ In vivo, adenovirus‐induced overexpression of miR‐24 promoted re‐endothelialization in balloon‐injured diabetic rats.^48^

#### 4.2.2 Modulation of c‐Jun N‐terminal kinase (JNK)

JNK has been shown to regulate vascular NRF2 expression and function upon diabetes. However, the role of JNK in control of NRF2 is controversial. The pioneer work by He et al showed that AGEs induced rapid phosphorylation of JNK in bovine aortic endothelial cells. Inhibition of JNK by SP600125 abrogated the expression of the NRF2 downstream antioxidant HO1,^49^ suggesting that JNK positively regulates NRF2 signalling. However, this study did not look into the impact of SP600125 on the expression of NRF2 and its various downstream antioxidant genes other than *Hmox1*. Additionally, the impact of HG on the activity of JNK and NRF2 was not studied. Therefore, the impact of JNK on vascular NRF2 expression and function under diabetes/HG conditions was still unclear. More recently, naringenin—a flavanone found in various plants—elevated NRF2/HO1 and inhibited HG‐ or free fatty acid–induced apoptosis of human umbilical vein endothelial cells (HUVECs).^50^ Naringenin phosphorylated AKT serine/threonine kinase (AKT) and JNK in HUVECs under the normal glucose condition and failed to increase HO1 protein level in the presence of SP600125 or the phosphoinositide 3‐kinase (PI3K) inhibitor Y294002, indicating that naringenin might activate NRF2/HO1 through activation of JNK and AKT.^50^

On the contrary, JNK was found by other groups to negatively regulate NRF2. C66—a novel curcumin analogue—has shown NRF2‐activating efficacy in both the aortas^51^ and kidneys^24^ of STZ‐induced diabetic mice. C66 was primarily identified to be an inhibitor of JNK.^51^ Inhibition of JNK by either C66 or SP600125 activated NRF2 expression and function and attenuated the diabetes‐induced aortic pathological injuries to a similar extent,^51^ suggesting that JNK negatively regulates NRF2 in the aorta. Moreover, a recent study showed that C66 predominantly targeted JNK2, as both *Jnk2* gene deletion and C66 treatment could similarly activate NRF2 and alleviate diabetes‐induced aortic oxidative stress, inflammation and fibrosis.^43^ Supporting these findings, our group found that SP600125 prevented DN through activation of NRF2, with enhanced expression of *Hmox1* and *Nqo1*.^52^ SP600125 hampered the HG‐induced phosphorylation of JNK and c‐Jun, repressing *Keap1* gene expression.^52^ This inhibitory impact of SP600125 on KEAP1 was viable because SP600125 induced remarkable nuclear translocation of NRF2.^52^ Supporting the inhibitory effect of SP600125 on* Keap1* gene expression, 36 c‐Jun‐binding sites were found between −3000 bp and −1 bp within the promoter region of the mouse *Keap1* gene.^52^ Luciferase reporter assay should be helpful to further confirm the regulatory effect of JNK on *Keap1* gene transcription.

As reviewed above, controversies exist in defining the role of JNK in NRF2 activity in the vasculature. These controversies may be owing to the differences between cell types (BACEs, HUVECs, whole aortas and mouse mesangial cells) and (or) disease models (stimulation with AGEs vs HG/hyperglycaemia). Further studies are needed to elucidate the regulatory effect of JNK on NRF2 in MCD.

#### 4.2.3 Transcriptional activation of *Nfe2l2*

Recently, NRF2 activation has been achieved in diabetes‐induced endothelial dysfunction at the transcription level. Sodium butyrate (NaB), a polyunsaturated fatty acid found in food, was primarily reported by our group to activate renal *Nfe2l2* gene expression in an STZ‐induced type 1 diabetic mice.^53^ We further found that NaB improved the diabetes‐induced aortic endothelial dysfunction in the wild‐type, but not *Nfe2l2* gene knockout, mice.^28^ Mechanistically, in HG‐treated aortic endothelial cells, NaB elevated *Nfe2l2* mRNA and protein levels without facilitating NRF2 nuclear translocation, an effect distinct from that of SFN. Further, NaB inhibited the activity of histone deacetylase (HDAC) and increased the occupancy of the transcription factor aryl hydrocarbon receptor and the co‐activator P300 at the *Nfe2l2* gene promoter. Moreover, the P300 inhibitor C646 completely abolished these efficacies of NaB.^28^ These results suggest that NaB prevented diabetes‐induced aortic endothelial dysfunction through HDAC/P300‐mediated transcriptional activation of *Nfe2l2*.

#### 4.2.4 Preservation of NRF2 protein

Preservation of NRF2 protein from degradation is another efficacious way to maintain cellular NRF2 protein level, facilitating NRF2 activation. MG132—a proteasome inhibitor—preserved NRF2 protein and attenuated aortic pathological injuries in OVE26 diabetic mice.^54^ MG132, administered 3 months starting from the age of 3 months old, was shown in this study to lower the diabetes‐provoked aortic levels of tumour necrosis factor‐alpha (TNF‐α), plasminogen activator inhibitor‐1, 3‐nitrotyrosine and 4‐hydroxynonenal. Moreover, MG132 ameliorated the diabetes‐induced thickening and structural derangement of the aortic wall.^54^ It is noted that the pathological aortic changes at the initiation of the treatment (3‐month‐old OVE diabetic mice) were not evident. MG132 should have yielded greater beneficial effects if administered at earlier stages of diabetes, since once formed, it is impossible to reverse the diabetes‐induced typical pathological features.^55^

#### 4.2.5 Inhibition of nuclear export of NRF2

##### Zinc (Zn)

Zn is an essential trace element that has antioxidant activity. Zn deficiency in endothelial cells enhanced inflammatory response and oxidative stress.^56^, ^57^ On the contrary, Zn supplementation benefitted the aortas of 3‐month‐old OVE26 diabetic mice,^58^ including the attenuation of aortic fibrosis, oxidative stress, inflammation, apoptosis and proliferation.^58^ Moreover, Zn elevated both mRNA and protein levels of *Nfe2l2*,^58^ suggesting that Zn might regulate *Nfe2l2* gene expression at the transcription or post‐transcription levels.

The same group performed a further study to investigate the effect of Zn supplementation on DN. In OVE26 type 1 diabetic mice, Zn induced renal AKT and glycogen synthase kinase‐3 beta (GSK‐3β) phosphorylation with a decrease in proto‐oncogene tyrosine‐protein kinase Fyn (Fyn), a nuclear exporter of NRF2.^59^ Although this mechanism still cannot explain the former Zn‐increased *Nfe2l2* mRNA level, this study showed AKT/ GSK‐3β/Fyn as a mechanism, at least in part, by which Zn activated NRF2. Given that Zn deficiency is prevalent in patients with both type 1 diabetes and type 2 diabetes,^60^ Zn supplementation has a potential in the management of MCD.

##### Hydrogen sulphide (H_2_S)

H_2_S, together with NO and carbon monoxide, is an important member of the family of gasotransmitters.^61^ Liu et al observed a decreased H_2_S production in the aortas of 28‐week‐old db/db mice and in HG‐treated endothelial cells.^62^ Exogenous administration of sodium hydrosulphide decreased the levels of adhesive and apoptotic factors, increased nuclear accumulation of NRF2 and the expression of *Sod* and *Cat,* and suppressed the excessive autophagy induced by oxidative stress.^62^ More recently, the same group showed an inhibitory effect of H_2_S on VSMC proliferation under hyperglycaemic condition via inhibiting mitochondrial fragmentation.^63^ It was indicated in another study that PI3K/AKT was involved in H_2_S activation of NRF2 in cerebral ischaemia/reperfusion injury,^64^ suggesting a possible way through which H_2_S induced nuclear accumulation of NRF2—a mechanism similar to that of Zn.^59^ However, another study showed that sodium hydrosulphide S‐sulfhydrated KEAP1 at cysteine‐151, and facilitated NRF2 nuclear translocation, protecting against senescence in mouse embryonic fibroblasts.^65^ It would be interesting to investigate whether H_2_S structurally inhibits KEAP1 in MCD.

##### Baicalin

Baicalin is the major component found in Scutellaria baicalensis root, exhibiting anti‐inflammatory activity.^66^ Baicalin was reported to restore the activity of hyperglycaemia‐impaired aortic NRF2 signalling in STZ‐induced diabetic mice.^66^ Mechanistically, baicalin reversed the HG‐induced dephosphorylation of AKT and GSK‐3β in HUVECs, leading to the inactivation of Fyn. This promoted nuclear localization of NRF2 and transcription of *HMOX1*, *NQO1*, *NQO2*, *CAT* and *SOD2*, ameliorating HG‐induced oxidative stress.^66^

### 4.3 Other mechanisms to activate vascular NRF2 antioxidant signalling

Several other mechanisms have been studied.

#### 4.3.1 Insulin‐like growth factor 1 (IGF‐1)

In a mouse model of vascular ageing, liver‐specific knockdown of IGF‐1 decreased vascular oxidative stress resistance by impairing NRF2 signalling.^67^ In the aortas of IGF‐1‐deficient mice, the expression of *Nfe2l2* and downstream glutamate‐cysteine ligase catalytic subunit, *Nqo1* and *Hmox1* was decreased.^67^ When challenged with HG, the expression of these NRF2 downstream genes was activated in the wild‐type, but not IGF‐1‐deficient, aortas.^67^ This study indicates that IGF‐1 positively regulates NRF2. IGF‐1 activation or overexpression in animal or cell models of MCD could be helpful to confirm IGF‐1 as an activator of NRF2.

#### 4.3.2 HO1

HO1 is a known downstream antioxidant of NRF2 and has shown beneficial effects on endothelial cells and animal models of vascular disease.^68^ Inhibition of HO1 resulted in HG‐mediated endothelial cell damage.^69^ Supplementation of bilirubin, a HO1‐derived metabolite,^68^ restored endothelial cell viability under HG condition.^69^ Given the robust protective effect of HO1, two points of view are indicated based on this study. Firstly, HO1 might also be controlled by other regulators in addition to NRF2. Secondly, although NRF2 targets multiple downstream antioxidant genes, HO1 might exert the most robust antioxidant efficacy in MCD. This view is supported by the finding that metallothionein, another NRF2 downstream antioxidant, provided approximately 50% protection against DN upon SFN‐induced NRF2 activation.^70^

In summary, a number of NRF2‐activating strategies, either KEAP1‐inhibiting or not, exhibited beneficial effects on MCD (Table 1), providing versatile approaches for the management of MCD.

**TABLE 1:** Effect of NRF2 activators on MCD

Activator	Target	NRF2 dependence	Dose and period	Model	Effect	Reference
SFN	KEAP1 protein	Not verified	0.5 mg/kg, 5 d/wk, 16 wk	High‐fat diet + STZ‐induced type 2 diabetic mice	Aortic NRF2, HO1 and SOD1 proteins↑; 3‐NT↓, 4‐HNE↓, TNF‐α↓, VCAM‐1↓, apoptosis↓, proliferation↓, tunica media thickness↓, collagen accumulation↓	32
Dh404	KEAP1 protein	Not verified	3, 10 or 20 mg/kg/d, 18 wk	STZ‐induced type 1 diabetic mice	Oxidative stress↓, TNF‐α↓, ICAM‐1↓, MCP‐1↓, atherosclerosis↓, at lower (3 or 10 mg/kg/d), but not higher (20 mg/kg/d), doses	38
DMF	KEAP1 protein	Yes (*Nfe2l2* knockdown by siRNA)	25 or 50 mg/kg/d in mice; 5‐50 µmol/L in VSMCs	Vitamin D3‐induced aortic calcification mouse model; calcification media‐cultured VSMCs	Calcification↓, expression of bone marker genes *Runx2*, *Oc* and *Alp*↓	40
tBHQ	KEAP1 protein	Not verified	50 mg/kg, every other day, for 6 wk; 5‐24 µmol/L, 1‐3 h in VSMCs	STZ‐induced diabetes in *ApoE* ^‐/‐^ mice; VSMCs and macrophages exposed to IL‐6 and IFN‐γ	Atherosclerosis↓, lesional macrophages↓, foam cell size↓, chemokine expression↓	^42^
MiR‐200a mimic	*Keap1* mRNA	Yes (aortas and ECs isolated from *Nfe2l2* gene knockout mice)	1 mg/kg, 3 times weekly 30 nmol/L, 48 h	STZ‐induced type 1 diabetic mice HG‐treated mouse aortic ECs	Aorta: expression of *Keap1*↓, NRF2 protein↑; mRNA levels of *Nqo1*↑, *Homx1*↑, *iNos*↓, *Vcam‐1*↓ and *Mcp‐1*↓; 4‐HNE↓; endothelial dysfunction↓ ECs: expression of *Keap1*↓, t‐NRF2↑, n‐NRF2↑; mRNA levels of *Nqo1*↑, *Homx1*↑, *Vcam‐1*↓ and *Mcp‐1*↓; levels of ROS↓ and MDA↓; NO production↑	45
RGFP‐966	HDAC3/miR‐200a/*Keap1* mRNA	Not verified	200 mg/kg, every other day, for 3 mo	OVE26 type 1 diabetic mice	Aortic fibrosis and inflammation↓; hepatic HDAC3↓, miR‐200a↑, *Keap1* expression↓, NRF2/FGF21↑	47
Adenovirus‐induced overexpression of miR‐24	MiR‐24/*Keap1* mRNA	Not verified		Balloon‐injured diabetic rats; HG‐stimulated VSMCs	Re‐endothelialization↑; NRF2, HO1, SOD, GSH expression↑, ROS and MDA↓	48
SP600125	JNK	Not verified	20 µmol/L, 24 h	AGE‐stimulated BAECs	*Hmox1* expression↓	49
C66 or SP600125	JNK	Not verified	5 mg/kg, every other day, 12 wk	STZ‐induced type 1 diabetic mice	Aortic JNK phosphorylation↓, 3‐NT↓, TNF‐α↓, PAI‐1↓, apoptosis↓, proliferation↓, tunica media thickness↓, collagen accumulation↓	51
Naringenin	JNK and AKT	Not verified	50 µmol/L, 72 h	HG‐cultured HUVECs	Phosphorylation of JNK and AKT↑, NRF2 and HO1 protein↑	50
MG132	Proteasome	Not verified	10 µg/kg, 12 wk	OVE26 type 1 diabetic mice	Aortic 3‐NT↓, 4‐HNE↓, TNF‐α↓, PAI‐1↓, TGF‐β↓, CTGF↓, tunica media thickness↓, collagen accumulation↓	54
NaB	HDAC	Yes (aortas isolated from *Nfe2l2* gene knockout mice)	5 g/kg/d, 20 wk 10 µmol/L, 48 h	STZ‐induced type 1 diabetic mice HG‐treated mouse aortic ECs	Aortic expression of *Nfe2l2*/*Hmox1*↑, oxidative stress and inflammation↓, endothelial dysfunction↓ HDAC activity↓, AHR, P300 and H3K9ac occupancy at *Nfe2l2* gene promoter↑, endothelial expression of *Nfe2l2*, *Hmox1* and *Nqo1*↑, oxidative stress and inflammation↓	28
Zn (ZnSO_4_)	Possibly AKT/Fyn	Not verified	5 mg/kg, every other day, 12 wk	OVE26 type 1 diabetic mice	*Nfe2l2* mRNA and protein↑, MT↑, 3‐NT↓, 4‐HNE↓, VCAM‐1↓, TNF‐α↓, TGF‐β1↓, CTGF↓, PAI‐1↓, COL4↓, tunica media thickness↓	58
H_2_S (NaHS)	Possibly PI3K/AKT Not indicated	Not verified Not verified	100 µg/kg, every other day, 12 wk in mice; 100 µmol/L, 48 h, in cells 100 µmol/L, 24 h, in cells	db/db type 2 diabetic mice; HG + palmitate‐cultured rat aortic ECs HG‐cultured VSMCs	ATP↑, respiratory complex activity↑, AMPK phosphorylation↓, autophagy↓, apoptosis↓, adhesive molecules↓ Proliferation and migration of VSMCs↓, mitochondrial fragmentation in VSMCs↓	62 63
Baicalin	AKT/GSK‐3β/Fyn	Yes (*Nfe2l2* knockdown by shRNA)	50 mg/kg/d for 4 wk 50 µmol/L	STZ‐induced type 1 diabetic mice HG‐treated HUVECs	Phosphorylation of AKT and GSK‐3β↑, Fyn‐mediated nuclear export of NRF2↓, CAT, HO1 and NQO1↑, oxidative damage↓, endothelial impairment↓	66
Bilirubin	HO1	Not verified	0.5 µmol/L	HG‐cultured bovine aortic ECs	Cell viability↑, 4‐HNE↓	69
IGF‐1	Not indicated	Not verified	None	HG‐cultured aorta segments of liver IGF‐1‐deficient mice	Serum IGF‐1↓; NRF2 signalling in HG‐cultured aorta segments↓, oxidative stress↑, apoptosis↑, endothelial dysfunction↑	67

Although DMF was tested in experimental models of vascular calcification, but not diabetes, it is listed in this table as calcification is a pathological feature of MCD. Abbreviations: 3‐NT, 3‐nitrotyrosine; 4‐HNE, 4‐hydroxynonenal; AHR, aryl hydrocarbon receptor; AKT, AKT serine/threonine kinase; *Alp*, alkaline phosphatase; AMPK, AMP‐activated protein kinase; *ApoE*−/−, atherosclerosis‐prone apolipoprotein E‐deficient; ATP, adenosine triphosphate; BEACs, bovine aortic endothelial cells; CAT, catalase; COL4, collagen IV; CTGF, connective tissue growth factor; Dh404, dihydro‐CDDO‐trifluoroethyl amide DMF, dimethyl fumarate; EC, endothelial cell; FGF21, fibroblast growth factor 21; GSH, glutathione; GSK‐3β, glycogen synthase kinase 3 beta; H_2_S, hydrogen sulphide; H3K9ac, acetylated histone H3 lysine 9; HDAC, histone deacetylase; HG, high glucose; HO1, haem oxygenase 1; HUVEC, human umbilical vein endothelial cells; ICAM‐1, intercellular adhesion molecule 1; IFN‐γ, interferon‐gamma; IGF‐1, insulin‐like growth factor 1; IL‐6, interleukin‐6; JNK, c‐Jun N‐terminal kinase; KEAP1, Kelch‐like ECH‐associated protein 1; MCP‐1, monocyte chemoattractant protein‐1; MDA, malondialdehyde; miR‐200a, microRNA‐200a; miR‐24, microRNA‐24; MT, metallothionein; n‐NRF2, nuclear NRF2; NaB, sodium butyrate; *Nqo1*, NAD(P)H dehydrogenase (quinone 1); NRF2, nuclear factor (erythroid‐derived 2)‐like 2; *Oc*, osteocalcin; P300, E1A binding protein P300; PAI‐1, plasminogen activator inhibitor‐1; PI3K, phosphoinositide 3‐kinase; ROS, reactive oxygen species; *Runx2*, runt‐related transcription factor 2; SFN, sulforaphane; shRNA, short hairpin RNA; SOD1, superoxide dismutase 1; STZ, streptozotocin; tBHQ, tert‐butyl hydroquinone; t‐NRF2, total cellular NRF2; TGF‐β, transforming growth factor beta; TNF‐α, tumour necrosis factor alpha; VCAM‐1, vascular cell adhesion molecule‐1; VSMC, vascular smooth muscle cell. Symbols: ↑, upregulation or activation; ↓, downregulation or inhibition.

## 5 NRF2‐ACTIVATING STRATEGIES BORROWED FROM DIABETES AND CARDIOVASCULAR COMPLICATIONS OTHER THAN MCD

NRF2 activation in diabetes and cardiovascular complications other than MCD, such as DN, cardiomyopathy and retinopathy, has implications on MCD.

### 5.1 Sirtuin 1 (SIRT1)

SIRT1 is an HDAC and benefits diabetes and complications.^71^ Antioxidant efficacy contributes to SIRT1’s protective effects.^71^, ^72^ In a mouse model of STZ‐induced diabetes, SIRT1 and NRF2 antioxidant signalling were less expressed in the kidneys of the diabetic mice compared with non‐diabetic control mice.^73^ Paeonol (PA), a single phenolic compound extracted from the root bark of Cortex Moutan, increased renal SIRT1 protein level and activated NRF2 antioxidant signalling in the diabetic mice.^73^ In HG and PA cotreated rat mesangial cells, knockdown of *Sirt1* abrogated the PA‐induced activation of NRF2 signalling and reversed the inhibitory effect of PA on the expression of pro‐inflammatory and pro‐fibrotic genes.^73^ These results indicate that SIRT1 positively regulates NRF2 antioxidant signalling in protection against DN. Activation of SIRT1 may thus have potential in the intervention of MCD. Confirming this speculation, our group has demonstrated the beneficial effect of SIRT1 activation in diabetes‐induced endothelial dysfunction.^74^, ^75^ We found that SRT2104—a potent activator of SIRT1—attenuated diabetes‐induced aortic oxidative stress, inflammation and endothelial dysfunction in STZ‐induced diabetic mice through inhibition of P53.^74^ Because P53 inhibits *Nfe2l2* expression and function,^26^, ^76^ SIRT1 may activate NRF2 via inhibition of P53.

P53 is a transcription factor that activates the expression of miR‐34a which targets *Sirt1* mRNA.^75^ By inhibiting SIRT1 in the presence of either the P53 inhibitor pifithrin‐α or the miR‐34a inhibitor, our other work found that inhibition of P53/miR‐34a improved diabetes‐induced endothelial dysfunction through activation of SIRT1.^75^ Taken together, these studies have found a SIRT1/P53/miR‐34a circuit that acts upstream of NRF2 antioxidant signalling and controls endothelial function under diabetes.

### 5.2 Mouse double minute 2 (MDM2)/P53

MDM2 is a suppressor of P53.^77^ As P53 is primarily known as a guardian of the genome coordinating cellular responses to genotoxic stress,^78^ *MDM2* is considered as an oncogene in tumours.^77^ However, under diabetic conditions, MDM2/P53 shows a different profile from that in carcinogenesis. We and others have found that P53 is elevated and activated upon diabetes, contributing to endothelial dysfunction,^75^, ^79^ nephropathy ^26^, ^80^ and cardiomyopathy.^81^ Moreover, renal *Mdm2* expression is inhibited in STZ‐induced diabetic mice.^26^ Interestingly, inhibition of MDM2 by nutlin3a activated P53 and generated DN‐like pathologies in the non‐diabetic healthy mice.^26^ Notably, inhibition of P53 by pifithrin‐α activated renal NRF2 signalling and attenuated renal injuries in the wild‐type, but not *Nfe2l2* gene knockout, diabetic mice.^26^ In HG‐treated mouse mesangial cells, P53 gene silencing completely abolished nutlin3a's inhibitory effect on NRF2 signalling. Together, these findings demonstrate that MDM2 controls NRF2 antioxidant signalling via inhibition of P53 in DN, providing MDM2 as a novel target.

### 5.3 Protein kinase C delta (PKC‐δ)

Oxidative stress contributes to diabetes‐induced pancreatic beta‐cell damage.^82^, ^83^ Glucagon‐like peptide‐1 receptor (GLP‐1) is a peptide released by the gastrointestinal tract in response to nutrients such as carbohydrates, proteins and lipids,^84^ stimulating insulin secretion in the presence of elevated blood glucose concentrations.

Kim et al investigated whether NRF2 is involved in the protective effect of GLP‐1 on oxidative stress‐induced beta‐cell apoptosis.^85^ In this study, NRF2 antioxidant signalling was activated by exendin‐4 (EX4)—an agonist of GLP‐1—in palmitic acid‐ or hydrogen peroxide–stimulated beta cells.^85^ NRF2 was required for the protective effects of EX4 on ROS production and insulin secretion, as these effects were abolished when *Nfe2l2* was knocked down.^85^ Mechanistic study revealed that EX4 phosphorylated PKC‐δ. By using PKC‐δ siRNA in the presence of EX4, PKC‐δ was found to mediate the EX4‐induced activation of NRF2. Hence, PKC‐δ might be a positive regulator upstream of NRF2 in beta cells under oxidative stress.

### 5.4 PKR‐like ER kinase (PERK) and thrombospondin 1

Endoplasmic reticulum (ER) stress and oxidative stress are important mediators of beta‐cell failure in diabetes.^82^ ER stress develops when the protein load in the ER exceeds the capacity of the organelle to handle proper protein folding. In response, the cell activated an adaptive mechanism namely unfolded protein response which is controlled by the ER transmembrane protein PERK.^86^ NRF2 was reported to be a direct substrate of PERK.^87^ PERK phosphorylates NRF2, leading to dissociation of NRF2 from KEAP1.^87^ Cunha et al showed that thrombospondin 1—a multimeric Ca^2+^‐binding glycoprotein—was able to activate PERK and NRF2 in the endoplasmic reticulum, protecting against palmitate‐induced death,^86^ providing PERK and thrombospondin 1 as potential candidates upstream of NRF2 in the management of MCD.

### 5.5 DJ‐1 (Parkinson disease protein 7, PARK7)

DJ‐1, also known as PARK7, stabilizes NRF2 by preventing association of NRF2 with KEAP1.^88^ In support of the positive regulatory effect of DJ‐1 on NRF2, a recent study found that the protein levels of DJ‐1 and NRF2/HO1 were increased in the kidneys of STZ‐induced Sprague Dawley diabetic (4, 8 and 12 weeks of diabetes) rats.^89^ The role of DJ‐1 in the regulation of NRF2 antioxidant signalling in MCD warrants further investigation.

In summary, several targets identified in diabetes and its vascular complications other than MCD might shed light on future studies for MCD, as summarized in Table 2.

**TABLE 2:** Drawing lessons from diabetes and cardiovascular complications other than MCD

Target	Approach	Dose and period	Model	Effect	Reference
SIRT1/NRF2	Paeonol	150 mg/kg, 6 times/wk 5, 10 and 20 μg/mL	STZ‐induced diabetic mice HG‐treated rat mesangial cells	Renal SIRT1↑; NRF2↑; SOD activity↑; MDA↓; ICAM‐1↓; FN↓; mesangial matrix index↓ SIRT1↑; NRF2, HO1, SOD1↑; FN and ICAM‐1↓	73
MDM2/P53/NRF2	Nutlin3a PFT‐α	10 mg/kg, or 4 wk 1.1 mg/kg, 3 times weekly, for 24 wk	Non‐diabetic healthy mice DN (STZ‐induced diabetic mice)	DN‐like renal pathologies↑; UACR↑; renal oxidative stress, inflammation and fibrosis↑; P53↑; expression of *Nfe2l2* and downstream *Hmox1* and *Nqo1*↓ UACR↓; renal pathologies↓; renal oxidative stress, inflammation and fibrosis↓; P53↓; *Nfe2l2* and *Hmox1* expression↑	26
GLP‐1/PKC‐δ/NRF2	Exendin‐4	10 nmol/L	Palmitic acid‐ or hydrogen peroxide–stimulated beta cells	NRF2 protein level↑; *GCLC* and *HO‐1* mRNA levels↑; beta‐cell dysfunction↓	85
PERK/NRF2	Lyophilized thrombospondin 1 or overexpression of thrombospondin 1	2 μg/mL	Palmitate‐induced beta‐cell death	PERK↑; NRF2‐dependent ARE activity↑; GSTM1↑; CAT↑; beta‐cell death↓	86
DJ‐1/NRF2	Not available	Not available	DN (STZ‐induced diabetic Sprague Dawley rats)	Renal DJ‐1↑; NRF2/HO1↑	89

Abbreviations: ↓, downregulation or inhibition; DJ‐1, Parkinson disease protein 7 (PARK7); DN, diabetic nephropathy; FN, fibronectin; GCLC, glutamate‐cysteine ligase catalytic subunit; GLP‐1, glucagon‐like peptide‐1 receptor; GSTM1, glutathione s‐transferase mu 1; MDM2, mouse double minute 2; PERK, PKR‐like ER kinase; PFT‐α, pifithrin‐α; PKC‐δ, protein kinase C delta; SIRT1, sirtuin 1; UACR, urinary albumin‐to‐creatinine ratio. Other abbreviations are the same as in Table 1. Symbols: ↑, upregulation or activation.

## 6 NRF2 ACTIVATORS IN CLINICAL TRIALS FOR VASCULAR COMPLICATIONS OF DIABETES

Although no NRF2 activator has been applied in clinical trials for MCD, the clinical trials of NRF2 activators used in diabetic complications other than MCD may provide clues for the future trials of MCD.

### 6.1 Bardoxolone methyl in treatment of DN

The most well‐known NRF2 activator tested in clinical trials for the treatment of diabetic complications is bardoxolone methyl. The initial application of bardoxolone methyl in treatment of type 2 diabetic patients with 3b‐4 chronic kidney disease (CKD) yielded promising beneficial outcome.^90^ The 20 patients with moderate‐to‐severe DN received bardoxolone methyl at 25 mg/d for 28 days, followed by a dose of 75 mg/d for additional 28 days.^90^ By the end of the study, bardoxolone methyl dose‐ and time‐dependently elevated estimated glomerular filtration rate and significantly reduced serum creatinine and blood urea nitrogen, accompanied by an increase in creatinine clearance.^90^ Additionally, bardoxolone methyl blunted the biomarkers of vascular injury and inflammation.^90^ It is noted that no severe side effect was observed in all the patients.^90^

The success in the initial clinical trial of bardoxolone methyl brought light to the management of DN. Bardoxolone methyl was applied in a much larger phase 3 clinical trial in type 2 diabetic patients with stage 4 CKD thereafter.^91^ However, the trial was terminated because of severe heart complications.^92^ It is speculated that bardoxolone methyl may pharmacologically induce acute sodium and fluid retention, and therefore increase blood pressure and result in heart failure in patients with advanced CKD, despite its improvement of kidney injury in some of the patients.^92^, ^93^ Thus, attention should be paid to the off‐target effects of a therapeutic in addition to its dose. Moreover, criticism has been raised regarding the use of the NRF2‐activating drug at an inappropriate stage of the disease.^55^, ^94^ Application of the antioxidant approach should be more beneficial at a much earlier stage of DN, as typical pathological features of DN are impossible to be reversed,^55^, ^94^ whereas prevention of DN by NRF2 activators starting from the initiation of diabetes has proven successful in numerous experimental studies.^24^, ^53^, ^70^, ^94^, ^95^, ^96^

The benefits earned and lessons learned from the use of bardoxolone methyl in the clinical trials of DN have a positive impact on the potential application of NRF2‐activating approaches in future clinical trials of MCD, in which special attention should be paid to off‐target effects and occasion for intervention.

### 6.2 Curcumin in treatment of diabetic microangiopathy

Curcumin is a natural compound that derives from turmeric.^97^ Meriva, a lecithinized formulation of curcumin, was used in a pilot study on the management of diabetic microangiopathy.^97^ Meriva was given to 25 patients with diabetic microangiopathy at 1 g/d, for 4 weeks, improving diabetic microangiopathy and reducing oedema in the skin of the patients.^97^ Larger clinical trials with more detailed design are needed to further test the effect of Meriva on diabetic microangiopathy.

Despite the beneficial effects of Meriva, it is still unknown whether Meriva functioned through activating NRF2, as curcumin targets multiple factors. It is needed to elucidate the molecular mechanism of Meriva, which may provide evidence for potential off‐target effects.

The poor bioavailability restricts curcumin from clinical application.^24^ C66, a novel analogue of curcumin with much lower effective dose demonstrated in animals, has anti‐inflammatory, antifibrotic and antioxidative effects, and protects against diabetic cardiomyopathy,^98^ DN^24^ and MCD.^51^ C66's protection was shown to be partially mediated by NRF2.^24^ Therefore, C66 has a potential for clinical use in the management of MCD, based on its advantage in bioavailability and the solid preliminary work.

## 7 CONCLUSIONS AND PERSPECTIVES

**FIGURE 2:**
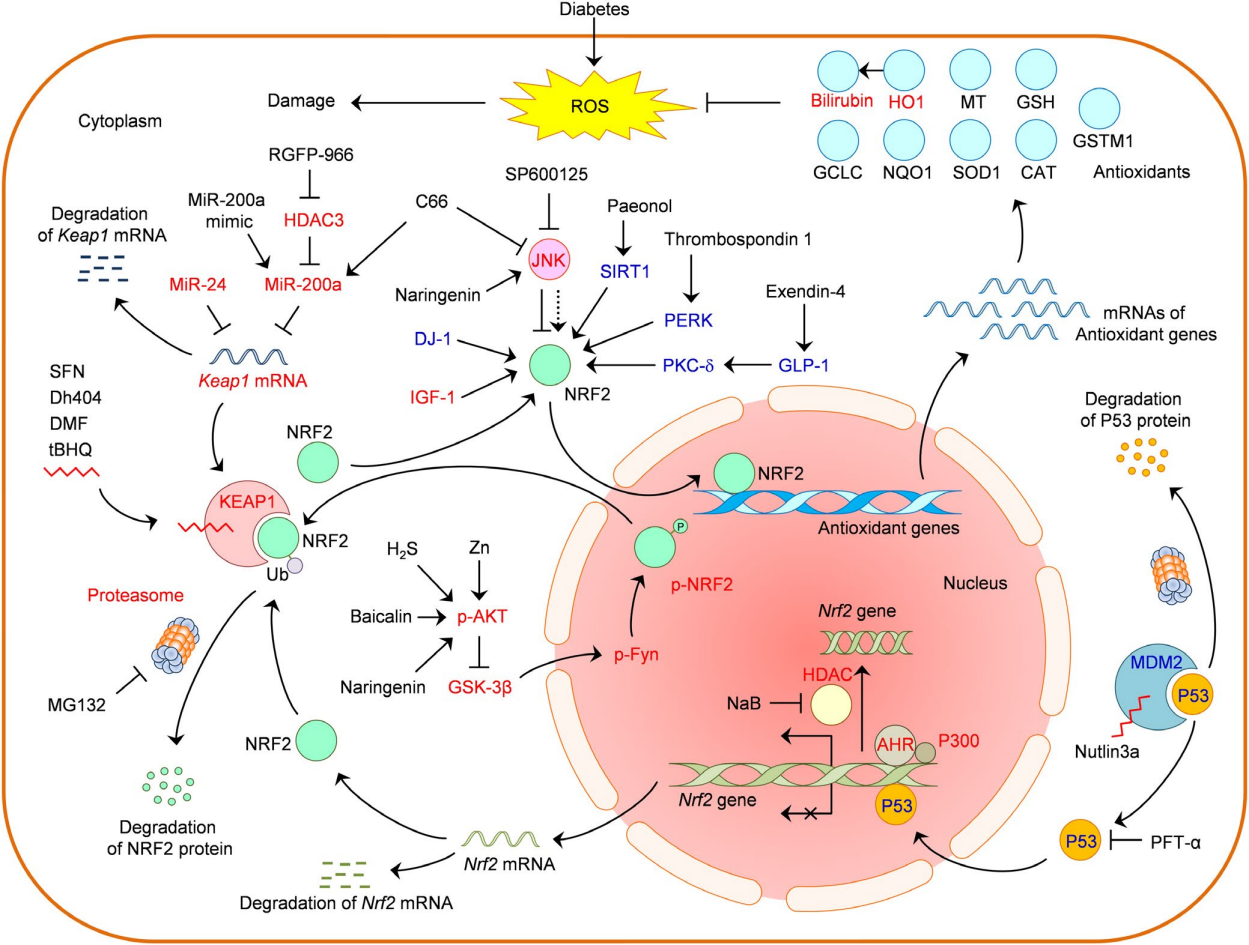
Schematic diagram for NRF2 activation in diabetes and its cardiovascular complications. Red characters, targets identified in macrovascular complications of diabetes (MCD); blue characters, targets identified in diabetes and cardiovascular complication other than MCD; symbols: **↓**, activation; _┴_, inhibition. AHR, aryl hydrocarbon receptor; CAT, catalase; Dh404, dihydro‐CDDO‐trifluoroethyl amide; DMF, dimethyl fumarate; DJ‐1, Parkinson disease protein 7 (PARK7); GCLC, glutamate‐cysteine ligase catalytic subunit; GLP‐1, glucagon‐like peptide‐1 receptor; GSH, glutathione; GSK‐3β, glycogen synthase kinase 3 beta; GSTM1, glutathione s‐transferase mu 1; H_2_S, hydrogen sulphide; HDAC, histone deacetylase; HO1, haem oxygenase 1; IGF‐1, insulin‐like growth factor 1; JNK, c‐Jun N‐terminal kinase; KEAP1, Kelch‐like ECH‐associated protein 1; MDM2, mouse double minute 2; miR‐200a, microRNA‐200a; miR‐24, microRNA‐24; MT, metallothionein; NaB, sodium butyrate; NQO1, NAD(P)H dehydrogenase (quinone 1); NRF2, nuclear factor (erythroid‐derived 2)‐like 2; P300, E1A binding protein P300; p‐AKT, phosphorylated AKT serine/threonine kinase 1; PERK, PKR‐like ER kinase; PFT‐α, pifithrin‐α; p‐Fyn, phosphorylated proto‐oncogene tyrosine‐protein kinase Fyn; PKC‐δ, protein kinase C delta; p‐NRF2, phosphorylated NRF2; ROS, reactive oxygen species; SFN, sulforaphane; SIRT1, sirtuin 1; SOD1, superoxide dismutase 1; tBHQ, tert‐butyl hydroquinone; Ub, ubiquitination; Zn, zinc

In recent years, the strategies for NRF2 activation have evolved from the canonical structural inhibition of KEAP1 protein to others such as microRNA‐induced inhibition of KEPA1 production, inhibition of proteasomal degradation of NRF2, inhibition of AKT/GSK‐3β/Fyn‐mediated nuclear export of NRF2 and modulation of HDAC/P300‐controlled *Nfe2l2* gene transcription (Figure 2, red characters). In these novel strategies for NRF2 activation, epigenetic mechanisms such as microRNAs^45^, ^48^ and histone modifications^28^, ^47^ have recently emerged as efficient approaches that show good potentials in the intervention of MCD. Other epigenetic mechanisms including circular RNAs, long non‐coding RNAs and DNA methylation also play important roles in the regulation of NRF2 antioxidant signalling.^99^, ^100^ These epigenetic mechanisms should provide new clues for future studies on NRF2 activation in MCD.

Moreover, regulators of NRF2 that were found in diabetes and cardiovascular complications other than MCD, including SIRT1, MDM2/P53, GLP‐1/PKC‐δ, PERK and DJ‐1, warrant further investigation for their roles in MCD (Figure 2, blue characters).

Taken together, NRF2 activation has a good potential in future clinical intervention of MCD. However, to date, the molecular mechanisms of NRF2 activation have not been fully understood. Further mechanistic studies are warranted. In addition, more novel and effective approaches should be developed as these will benefit the population with diabetes and MCD.

## CONFLICTS OF INTEREST

None.

## AUTHOR CONTRIBUTION

**Junduo Wu:** Data curation (lead); Investigation (equal); Writing‐original draft (equal); Writing‐review & editing (equal). **Xiaodan Sun:** Data curation (lead); Investigation (equal); Writing‐original draft (equal); Writing‐review & editing (equal). **Ziping Jiang:** Data curation (equal); Funding acquisition (equal); Investigation (equal); Writing‐review & editing (equal). **Jun Jiang:** Funding acquisition (equal); Investigation (supporting); Writing‐review & editing (equal). **Linlin Xu:** Funding acquisition (equal); Investigation (supporting); Supervision (equal); Writing‐review & editing (equal). **Ao Tian:** Investigation (supporting); Writing‐review & editing (equal). **Xuechun Sun:** Investigation (supporting); Writing‐review & editing (equal). **Huali Meng:** Investigation (supporting); Writing‐review & editing (equal). **Ying Li:** Funding acquisition (equal); Investigation (supporting); Supervision (equal); Writing‐review & editing (equal). **Wenlin Huang:** Investigation (supporting); Supervision (equal); Writing‐review & editing (equal). **Ye Jia:** Investigation (supporting); Supervision (equal); Writing‐review & editing (equal). **Hao Wu:** Conceptualization (lead); Data curation (lead); Funding acquisition (lead); Investigation (lead); Project administration (lead); Supervision (lead); Writing‐original draft (lead); Writing‐review & editing (lead).
